# Framing and the health policy process: a scoping review

**DOI:** 10.1093/heapol/czv128

**Published:** 2016-02-11

**Authors:** Adam D Koon, Benjamin Hawkins, Susannah H Mayhew

**Affiliations:** Department of Global Health and Development, London School of Hygiene and Tropical Medicine, London, UK

**Keywords:** Frames, ideas, health policy, policy process, scoping review

## Abstract

Framing research seeks to understand the forces that shape human behaviour in the policy process. It assumes that policy is a social construct and can be cast in a variety of ways to imply multiple legitimate value considerations. Frames provide the cognitive means of making sense of the social world, but discordance among them forms the basis of policy contestation. Framing, as both theory and method, has proven to generate considerable insight into the nature of policy debates in a variety of disciplines. Despite its salience for understanding health policy debates; however, little is known about the ways frames influence the health policy process. A scoping review using the Arksey and O’Malley framework was conducted. The literature on framing in the health sector was reviewed using nine health and social science databases. Articles were included that explicitly reported theory and methods used, data source(s), at least one frame, frame sponsor and evidence of a given frame’s effect on the health policy process. A total of 52 articles, from 1996 to 2014, and representing 12 countries, were identified. Much of the research came from the policy studies/political science literature (*n* = 17) and used a constructivist epistemology. The term ‘frame’ was used as a label to describe a variety of ideas, packaged as values, social problems, metaphors or arguments. Frames were characterized at various levels of abstraction ranging from general ideological orientations to specific policy positions. Most articles presented multiple frames and showed how actors advocated for them in a highly contested political process. Framing is increasingly an important, yet overlooked aspect of the policy process. Further analysis on frames, framing processes and frame conflict can help researchers and policymakers to understand opaque and highly charged policy issues, which may facilitate the resolution of protracted policy controversies.

**Key Messages**

- Framing offers key insights into understanding the nature of political debate by providing an explanation of both structure and agency in the policy process.
- Despite in-depth knowledge on a few key issues, little is know about the ways in which frames affect the health policy process.
- By following best practices, framing researchers can usefully interpret the forces that shape policy and strengthen the body of existing scholarship.

## Introduction

Tanks and divisions, and dollars and cents, you know all those things obviously make a difference, but ideas are the most powerful thing on Earth.—President Barack Obama, CBS 60 min (TV), 28 March 2014

The health policy arena is characterized by a number of highly charged ideological positions over a vast array of issues. In the field of public health, concepts such as ‘universal health coverage’ or ‘health workforce strengthening’ evoke particular value systems, courting public debate (Koon and Mayhew 2013). Similarly, technological innovation in biomedicine, the complexity of public and private financing arrangements, and the elaborately varied workforce, help to create a highly contested policy domain in which policy change is often incremental and slow (Béland 2010). New fields such as Health Policy and Systems Research (HPSR) have arisen to meet the growing demands of policymakers, researchers and practitioners for research that helps solve the problems of health systems in low- and middle-income countries (LMICs) (de Savigny and Adam 2009). Understanding the policy process is a central concern in this context because actors are often unsure what causes the rise and fall of certain ideas (Shiffman 2009). Furthermore, to understand how to respond effectively to policy challenges, actors need to know the nature of problematic situations and how specific actions generate particular policy responses (Fischer 2003). In this way, policy analysis can potentially help resolve protracted policy controversies (Schön and Rein 1994) and further the collective goal of sustainable health systems strengthening.

As a coherent body of scholarship materializes, HPSR researchers have increasingly pointed to conceptual and analytical shortcomings within the existing body of LMIC policy research (Walt *et al.* 2008; Walt and Gilson 2014). This includes research with little reference to methodological design, scarce use of established policy analysis theory, a lack of explanatory focus and a paucity of studies that ‘apply forms of analysis (such as discourse analysis) that consider the role of language, rhetorical argument and stories in framing policy debate’, (Gilson and Raphaely 2008). These shortcomings leave us with a fragile understanding of the policy process and the political forces that create policy change (de Leeuw *et al.* 2014). Moreover, the HPSR literature often fails to provide insight into how and why proposed policies are supported, dismissed or overlooked (Gilson and Raphaely 2008; Shiffman 2009; Berlan *et al.* 2014). For this reason, HPSR scholars have called for more research on the health policy process in order to understand the clash of values that determines the mix of policy considerations and collectively contributes towards the achievement of shared health objectives (Bennett *et al.* 2011; Sheikh *et al.* 2011). In order to answer these calls, HPSR scholars are looking to other disciplines for methodological inspiration (Gilson *et al.* 2011).

Outside the health literature, the field of policy studies has seen the emergence of interpretative modes of analysis, which reject the narrow, rationalist assumptions of ‘mainstream’ political science, which takes its epistemology and methodological lead from the natural sciences (Fischer and Forester 1993; Fischer and Gottweis 2012). Against this, interpretivists argue that there are fundamental differences between the social and the physical realm (Rabinow and Sullivan 1987). Moreover, different forms of knowledge are possible in each domain, which in turn necessitate different methodological approaches by the researcher (Yanow and Schwartz-Shea 2006). The difference between the social and physical worlds relates principally to the focus of the social sciences on reflexive human agents (Rabinow and Sullivan 1987). Humans, unlike other physical objects, are involved in a constant process of interpreting and assigning meaning to the events, processes, objects and actions they experience; meanings which morph and change through social interactions with other agents (Schutz 1962). Thus, humans are engaged and embedded in the social construction of multiple, but equally legitimate, interpretations of social reality, which are open to change and reinterpretation (Berger and Luckmann 1967).

Approaches to policy analysis that draw on a constructivist epistemology, often employ interpretive methods to accommodate the tacit role that values, beliefs and feelings play on our ability to impart meaning to social action (Yanow 1996). These policy analysts frequently argue that politics is simply the struggle over ideas, their meanings and competing interpretations about what is right (Stone 2012). The ability to communicate meaning and reach a shared sense of understanding underscores the salience of language and symbolic action in the policy process (Edelman 1988). Furthermore, policy analysts seek to understand behaviour and social practices in terms of goals and values, rather than provide causal explanations of complex social phenomena (Fischer 2003).

This article assesses the scope of the current body of framing scholarship on the health policy process. This review represents an initial attempt to harness a body of work on interpretive policy analysis, specifically framing research, to understand more about the ways in which ideas influence the policy process. In so doing, the authors hope to bridge the health policy and broader policy studies literatures. This review aims to demonstrate the potential value of constructivist and interpretative approaches to policy analysis for the domain of health policy and practice. It highlights the ways in which researchers outside of the health domain use theory to gain a better understanding of contestation and change in the policy process. In the following section, theory is introduced and a scoping review is presented using a well-established framework (Arksey and O’Malley 2005). This literature is then critically appraised, highlighting the insight gained through framing analyses and the relative merits/shortcomings of such an approach. Potential lines of enquiry are suggested to help position HPSR as an important vehicle for furthering our understanding of the policy process in the health sector.

## Theory

Policy scholarship on framing has evolved from a research tradition that focuses on the primacy of ideas in explaining policy dynamics and variation (John 2012). This contrasts with other theories of the policy process including those oriented around interests (Bachrach and Baratz 1962; Mills 1956; Dahl 1961), institutions (March and Olsen 1984), metaphors that blend elements of each (Baumgartner and Jones 1993; Sabatier and Jenkins-Smith 1993a,b; Kingdon 1984) and analytical eclecticism (Sil and Katzenstein 2010). The influence of ideas on the policy process was vividly captured by Weber (1946), ‘…“ ideas” have, like switchmen, determined the tracks along which action has been pushed by the dynamic of interest,’. As causal beliefs, ideas shape our understanding of policy problems, anchor our preferences, express our goals, and inject a sense of purpose to political debate (Béland and Cox 2011). Ideas present the policy researcher with an interesting entry point for understanding policy by providing clear linkages to institutions (Schmidt 2011), conceiving of interests as social constructions (Hay 2011) which leaves room to account for irrational behaviour (Kahneman 2011) in the policy process, and by capably handling abstract concepts such as power and domination (Fraser 1989; Jenson 1989; Lieberman 2002). Moreover, the flexibility of ideational approaches allows policy analysts to account for the ways in which ‘…thoughts, emotions and desires, as well as interests, are held in delicate and fluid balance with one another’ (Béland and Cox 2011:11).

The ‘frame’ is considered to be an optimal unit of analysis in ideas-based policy research, as it constitutes either a package of ideas (Gitlin 1980) or a central organizing idea (Gamson and Modigliani 1987). Framing research gained currency through the early work of anthropologist Gregory Bateson and sociologist Erving Goffman in the 1950s and 1970s, respectively (Bateson 1972; Goffman 1974). In his seminal work ‘Frame Analysis’, Goffman defined interpretive frames as a principle of organization ‘which governs the subjective meaning we assign to social events’ (Goffman 1974:10–11). Frames have been used to organize meaning and concepts in a wide variety of settings, from its psychological origins in the idea of ‘schemata’ (Bartlett 1932) to linguistics (Tannen 1993; Lakoff 2004, 2006), social movements research (Gitlin 1980; Snow *et al.* 1986; Snow and Benford 1988), communication and media studies (Tuchman 1978; Gamson *et al.* 1992; Entman 1993; Iyengar 1991), political psychology (Chong and Druckman 2007b), the study of social problems (Gusfield 1981), health communication (Rothman and Salovey 1997), behavioural economics (Tversky and Kahneman 1981; Kahneman and Tversky 1984) and policy studies (Schön and Rein 1994; Van Hulst and Yanow 2014). Common to most of these interpretations is the constructivist premise that an issue in society can be viewed in myriad ways and cast so as to imply multiple values and considerations (Berger and Luckmann 1967). As such, framing is a dynamic process through which those who produce and receive frames make sense of ideas by interpreting them through other available social, psychological and cultural concepts, axioms and principles (Fischer 2003). Thus, frames provide, ‘meaning to an unfolding strip of events’ (Gamson and Modigliani 1987). But frames are much more than packages of meaning. Frames can also be ‘weapons of advocacy’ (Weiss 1989).

In policy analysis, framing is largely situated in the post-positivist literature that uses interpretive and critical approaches to analyse policymaking as a contested meaning-making enterprise (Fischer 2003). Within this literature, framing in both form and function is closely related to the concept of metaphor (Lakoff and Johnson 1980; Schön 1993), causal storylines (Stone 1989), narrative (Roe 1994), policy problems (Gusfield 1981; Spector and Kitsuse 1987) and discourse (Laclau and Mouffe 1985; Fairclough 1992; Howarth 2000). These concepts underscore the importance of language and symbolic representation in the policy process (Edelman 1977, 1985, 1988; Elder and Cobb 1983; Gamson 1992). Following this approach, critical or interpretive policy analysts attempt to ‘…understand how, under what conditions, and through which processes specific frames emerge and are maintained’ (Hawkins and Holden 2013). In this way, the analyst favours knowledge claims of subjective understanding over objective truths, to the extent that interpretation provides a reasonable explanation of human behaviour, including evidence use, argumentation and persuasion in the policy process (Majone 1989).

In the disciplines of political psychology and communication, the concept of framing is deployed to analyse public preference formation. Within this literature, framing draws heavily on the field of behavioural economics (Kahneman and Tversky 1979; Tversky and Kahneman 1981) to look at the cognitive basis for decision making (Druckman 2004). Frames, in this context, are heuristic devices which shape our understanding and evaluation of the world around us based upon the extent to which they are cognitively available, accessible, applicable and appropriate (Druckman 2011). Emphasis (or issue) frames represent cognitively coherent dimensions of an issue that are assigned weights in preference formation (Druckman 2011; Scheufele and Iyengar 2012). In contrast, ‘equivalency’ or ‘valence’ frames represent value-based evaluations within a single set of dimensions, causing a frame to be portrayed either negatively or positively (Levin *et al.* 1998). This literature distinguishes these cognitive frames from their communicative forms, by drawing on research from the field of political communication (Scheufele and Iyengar 2012). When communicative frames affect individual cognitive frames a ‘framing effect’ has occurred, which allows the researcher to analyse the rhetorical basis for public attitudes (Druckman 2011) and the effectiveness of rhetorical strategy (Jerit 2008, 2009). In media studies, framing effects are carefully distinguished from the related processes of agenda-setting and priming (Scheufele and Tewksbury 2007). A frame’s ‘strength’, akin to the concept of ‘frame resonance’ from social movements research (Snow and Benford 1988), seems to play a more crucial role in determining the size of the effect in competitive environments than a frame’s repeated usage (Chong and Druckman 2007a; Druckman 2010). In this way, the literature on framing from political psychology and political communication has become influential in exploring social and political phenomena such as voter behaviour and public opinion formation (Druckman *et al.* 2009).

As noted, the concept of framing is used in related, yet distinct, ways in other academic disciplines. Within these different approaches, frames are seen to function in a variety of ways. In Goffman’s conception, frames balance structure and agency because our world is framed by events and experiences and yet we actively frame events and experiences (Gamson *et al.* 1992). Both overtly and covertly, frames highlight certain aspects of a problematic situation, while obscuring others in order to define problems, diagnose causes, make moral judgments and suggest remedies (Entman 1993). This is important in the policy world because frames determine what the actors in the policy community will consider the facts to be and how competing problem definitions lead to normative prescriptions for action (Rochefort and Cobb 1994). Framing precludes certain policy responses, identifying legitimate participants through political discourse and galvanizing coalitions of interest (Schattschneider 1960). Moreover, when comparing multiple perspectives on how to address a particular problem, the problem itself may change through framing (Fischer 2003). Additionally, actors may try to strategically change the problem by reframing a policy dilemma to incorporate a broader array of interests and potentially free the decision-making process from the gridlock of conflicting frames (Schön and Rein 1994). This highlights the transformative nature of discourse in the sense that ‘frames in communication’ influence ‘frames in thought’ (Druckman 2011).

Because frames serve multiple purposes, scholars from a variety of disciplines have attempted to classify them at various ‘levels of abstraction’ (Gamson *et al.* 1992). As mentioned previously, frames can be classified based on whether they define, diagnose, judge or prescribe (Entman 1993). Similarly, other scholars suggest that diagnostic, prognostic and motivational collective action frames are requisite for the emergence and mobilization of social movements (Snow and Benford 1988). As highlighted earlier, some researchers differentiate between communicative frames and cognitive frames, which can be classified into emphasis and equivalency frames (Druckman 2011). Equivalency frames can be further ordered into risky choice, attribute and goal frames (Levin *et al.* 1998). Others draw distinctions between rhetorical and policy action frames, which can be further subdivided into metacultural, institutional and policy frames (Schön and Rein 1994). This is analogous to linguist classification according to a frame’s depth such as values frames (deep), broad issue domain frames (intermediate) and detailed descriptive issue frames (shallow) (Lakoff 2006; G. Lakoff, personal communication as cited in Dorfman *et al.* 2005). Other linguists classify the components of frames into four structural dimensions of a greater news discourse, including their syntactical, script, thematic and rhetorical structures (Pan and Kosicki 1993). Similarly, a brand of media content analysis identifies the linguistic artifacts of a given frame, and allows the analyst to organize them into a ‘signature matrix’ (Gamson and Lasch 1983). Together, this array of frames, framing processes and approaches to frame analysis provide a fertile body of knowledge to cultivate insights into previously unexplored policy domains.

## Methods

This article used scoping review methods developed by Arksey and O’Malley (2005) to characterize, the full range of framing research in health policy, its content, and any potential gaps that require further exploration. Scoping review methodology has been discussed in key methodological texts (Petticrew and Roberts 2006; Grant and Booth 2009; Rumrill *et al.* 2010; Aveyard 2014) and is increasingly used in HPSR (Mitton *et al.* 2009; Brien *et al.* 2010; Ridde and Morestin 2011). This approach was selected because of its emphasis on flexibility, relying on an abductive logic of enquiry, and its bias towards narrative driven summation (see Table 1). Like all research, and particularly qualitative research, this approach is interpretive in nature. The Arksey and O’Malley framework is presented as an iterative, qualitative review with five distinct stages, each of which is described in greater detail below: (1) Identifying the research question (2) Identifying relevant studies (3) Study Selection (4) Charting the data (5) Collating, summarizing and reporting the results.

**Table 1.** Comparison of scoping vs systematic reviews

Systematic review	Scoping review
• Narrow research question & parameters	• Research question usually broad
• Pre-defined Inclusion/exclusion	• *Post hoc* Inclusion/exclusion possible
• Quality filters often included	• Quality not an initial concern
• Data extraction highly detailed	• Data extraction not required
• Quantitative synthesis typically	• Qualitative synthesis typically
• Structured assessment, with quality appraisal, to answer focused research question	• Identification of key issues and knowledge gaps in a body of literature

Adapted from Brien *et al.* (2010)

The research question emerged gradually through the review process. This became the following: ‘What is known from the existing literature about the influence of frames on the health policy process?’ This question drew important distinctions that precluded the exclusion of salient framing research from other sectors and framing research that does not illustrate the effects of frames on the policy process itself. This is important because framing is commonly used to describe a variety of research endeavors that explore the effects on individual actors and behaviours, but doesn’t always show how their contested interpretations shape policy design, especially in the health sector. Therefore, our initial decision was to include only articles that explicitly state a frame, its construction, its sponsor, and the ways in which it influenced the policy process in the health sector.

A review of the peer-reviewed literature was conducted for original research articles that used some form of frame analysis within the broad domain of health. Nine different social science and health databases were searched in June 2014 with search criteria that incorporated the term ‘fram*’ combined with the term ‘health policy’, excluding the term ‘framework’. This search strategy proved impractical as it yielded too many studies that referred to lay conceptions of ‘framing’ while not representing a coherent body of framing research. To produce a more representative body of work, the search was repeated using the search term ‘framing’ combined with ‘health policy’, both of which had to be present in at least the abstract of an article. No time or language restrictions were placed on any of the databases. See Table 2 for a list of databases with their corresponding search terms and number of hits. In addition to the database search, we used Google and Google Scholar search engines to identify sources not included in electronic databases. Finally, we conducted a hand-search of four health policy journals that publish framing research on occasion, including: *Health Policy and Planning, Social Science and Medicine, Health Policy, and Journal of Health Politics, Policy and Law.*

**Table 2.** Search terms

Database	Search term	Hits	w/o duplicates
**ProQuest**	‘Health Policy’ AND framing	315	
**PsychInfo**	exp (gov. policymaking/or exp (healthcare policy) or exp (policy making) or exp (health policy) AND exp (framing effects / framing mp.	419	356
**Pubmed (Med-line)**	‘policy’ [MeSH Major Topic] AND framing	140	67
**EMBASE**	‘health policy’ AND ‘framing’	317	150
**EBSCO Academic Search Premiere**	Health Policy AND framing	259	142
**Web of Science**	‘health policy’ AND framing	204	131
**EBSCO SSFT**	Health Policy AND framing	58	19
**CINAHL**	txt(Health Policy) AN ab(framing)	62	11
**JSTOR**	‘health policy’ AND ab(framing)	61	40
**TOTAL**			1231

**Figure 1.**
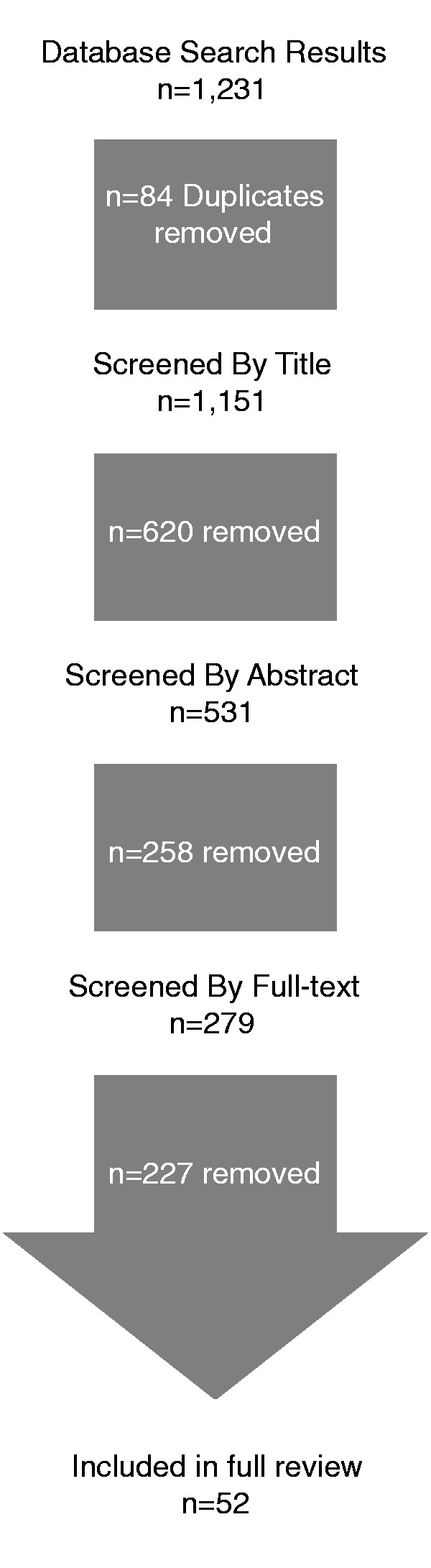
Scoping review flow diagram

Articles were excluded sequentially by ADK based on their title, abstract and full-text. Co-authors BH and SHM were consulted for questionable exclusions. Articles that alluded to framing, language, metaphor, discourse and its effects on health policy issues were included in the title review. During abstract review, an article was required to have the word ‘frame’ or ‘framing’ present in the abstract as well as a vague health policy issue to be included. Finally in the full-text review, all articles were reviewed to assess the extent to which frames, a frame articulator, and a contested policy process was explicitly represented. Because our conception of the policy process was oriented around established notions of contestation and deliberation, reference to a lineage of framing theory served as additional inclusion/exclusion criteria. In this way, the review attempted to draw from the wider pool of non-health policy issues, to assess the various ways in which frame conflict and change shapes the policy process. See Figure 1.

Akin to data extraction, a process of data ‘charting’ was initiated by ADK, consistent with the Arksey and O’Malley framework. The charting fields were developed in consultation with co-authors BH and SHM. A master table was created that included article details, corresponding research traditions, epistemology, theory employed, methodological approach, data sources, health and policy themes, frames, frame sponsor and the extent to which contestation had an effect on the policy process. Though this was systematic, the process of charting involved some degree of interpretation on the part of the investigators to classify various themes such as research traditions and the epistemology represented in each article. The investigators made no claims of objectivity in judging whether or not an article presented contestation or adequately showed an affect on the policy process. This reflects a growing distinction between systematic and scoping reviews and was in fact one of the motivations for relying on the Arksey and O’Malley framework.

The final stage of the scoping review process involved collating, summarizing and reporting the findings, as described in greater detail below. A descriptive analysis of collated articles by field was reported and general trends were identified. The findings were summarized with an emphasis on the scope of existing knowledge and an eye to what remains unclear from the body of research. Further suggestions about the conduct and import of framing research in the health sector are discussed and limitations of such an approach are considered, below.

Author reflexivity is important because interpretation and narrative summation are central to the Arksey and O’Malley scoping review framework. All three authors are social scientists with experience conducting qualitative research. The authors’ disciplinary training and in-depth knowledge of interpretive policy analysis, particularly frame-critical approaches, have shaped their understanding of the health policy process and the role of framing more generally. Though we make no claims to objectivity, we have attempted to provide a fair and balanced account of the various strands of framing research and their representation in the health policy literature. Thus, the term ‘framing research’, as employed in this article, is expansive and unattached to a specific epistemology. Rather the use of the term is consistent with the principles of analytical eclecticism (Sil and Katzenstein 2010).

## Results

A large number of framing studies were conducted on health policy issues, predominately from the social sciences. A total of 1231 articles were returned from the initial search. From these, a title review, supplemented with cursory abstract review, further narrowed the number of articles to 279. The exclusion/inclusion criteria were applied in the next round of reviewing to all abstracts and when necessary, a cursory full-text review. Finally, 52 articles were determined to represent framing research in which the following was explicitly stated: theory and methods used, data source, at least one frame, frame sponsor and some evidence of a given frame’s effect on the health policy process. See Appendix for an overview of 52 articles, which are characterized in greater detail below.

The number of relevant research articles is increasing in volume and geographic coverage. Articles ranged from 1996 to 2014. The number of relevant research articles is increasing rapidly (1990s, *n* = 3, 2000s, *n* = 17; 2010s, *n* = 32). Studies were reported from several countries (*n* = 12), with the USA representing the highest number of articles (*n* = 15). There were a handful (*n* = 4) of cross-country comparative studies and 12 studies focused on global framing of health policy issues. Although the majority were research articles from peer-reviewed journals, several doctoral theses/dissertations were included (*n* = 5). A large framing research project with a summary article (McInnes *et al.* 2012) and individual articles (*n* = 6) packaged as a journal supplement were included and counted individually. Two articles represent obesity framing research (Saguy and Riley 2005; Kwan 2009) from larger bodies of work represented in separate books (Kwan and Graves 2013; Saguy 2013). The books themselves were not included as the peer-reviewed articles were considered sufficient. Conversely, a book on children’s health insurance (Sardell 2014) was included in the review because framing research within the book was not found in the peer-reviewed journal literature.

Framing research varied across social science disciplines, epistemology and drew from multiple framing theories. Of the 52 articles included in this review, 25% (*n* = 13) were classified as health policy research endeavors. The majority of framing research on health has been conducted in the following research traditions: policy studies (*n* = 14), political science (*n* = 4), sociology (*n* = 9), international relations (*n* = 8), psychology (*n* = 2) and media studies (*n* = 2). The majority of articles were classified as operating from a constructivist epistemology (*n* = 42). The remaining articles used positivism (*n* = 2), realism (*n* = 1) or used epistemologies that were difficult to identify (*n* = 4). To be included, an article had to draw on established framing theory (as described earlier). Many articles drew from theoretical advances in the Interpretive/Critical Policy Analysis tradition (*n* = 13). Nearly all articles signaled Goffman (1974) as the theoretical origin, though Entman (1993), Gamson (1992) and Benford and Snow (2000) were frequently cited as well.

Framing research relied on multiple data sources and covered a range of health topics. Nearly all articles made reference to some degree of document review. The majority used published texts (*n* = 34), such as newspapers or government reports, often analysed by a variant of content analysis. Research also relied on in-depth interviews with key informants (*n* = 22). Several health issues were covered by the scope of research, including infectious disease (*n* = 10), substance misuse (*n* = 9), non-communicable diseases (NCDs) (*n* = 6), reproductive and sexual health (*n* = 5), access to medicines (*n* = 4), environmental health (*n* = 3) and others. Of the infectious disease studies, 60% (*n* = 6) were studies that focused on HIV/AIDS, 30% (*n* = 3) focused on various aspects of influenza and 10% (*n* = 1) concerned SARS. Of the studies categorized as substance misuse, 55.6% (*n* = 5) were tobacco studies, 33.3% (*n* = 3) were alcohol studies and 11.1% (*n* = 1) concerned injection drug use. The NCDs studies were split between cancer (*n* = 3) and obesity (*n* = 3). In sum, a wide range of data sources and health topics were covered with some issues (i.e. HIV/AIDS and tobacco control) better represented than others.

Numerous frames were presented with variable interpretations of the concept. The number of frames represented in a single research project ranged from 44 (Andress 2007) to one (Abraham 2011; Kamradt-Scott and McInnes 2012). The term ‘frame’ was used in different ways. Some articles referred to frames when describing packages of ideas that align with a particular value base (Esmail and Kohler 2012; Parkhurst 2012; Oronje 2013). Other articles used the idea of framing to refer to the construction of social problems (Kolker 2004; Studlar 2008; Blackman *et al.* 2012). This included contestation over diverging interpretations or portrayals of both the causes and solutions to specific policy dilemmas (Garvin and Eyles 2001; Driedger and Eyles 2003; Daw *et al.* 2014). Other articles focused on the linguistic construction of frames, akin to Lakoff’s work on metaphor (Ibrahim 2007; Dodge 2008). Finally, articles used the term ‘frame’ synonymous to ‘argument’, where policy dilemmas are structured by competing claims about what is fair and what is right (Moret-Hartman *et al.* 2006).

Similar to the multiple uses of the term frame, authors located frames at varying degrees of abstraction ranging from broad values (Johnson 2010; Rasmussen 2011; Esmail and Kohler 2012; Reubi 2012) to specific policy positions (Redington 2009; Fogarty and Chapman 2011, 2012; Paterson and Marshall 2011; Parkhurst and Vulimiri 2013). This corresponds to various strands of framing research including Schön and Rein’s (1994) ladder of policy action frames (Iannantuono and Eyles 2000; Firbank 2011), Benford and Snow’s (2000) classification of collective action frames (Frickel 2004; Noy 2009) and Gamson and Lasch’s (1983) signature matrix (Kwan 2009; Jenkin *et al.* 2011; Tynkkynen *et al.* 2012). This was sometimes difficult to identify, as many articles failed to specify the theoretical basis for their specific interpretation of frames. Few articles distinguished between different types of frames or the ability of various ideas to overlap and correspond to multiple legitimate frames constructed at various levels of abstraction.

Diverse policy stakeholders were identified as frame sponsors, responsible for creating, supporting, or opposing contested policy frames. Though most articles presented at least one group of frame articulators from the public sector, frame articulators lacked many unifying characteristics and were often specific to the issue or focus of the research project. Most articles provided a strong account of policy contestation (*n* = 40) while others provided some evidence of conflict (*n* = 8), and a few provided very little (*n* = 3). Contestation was context specific, but frequently represented deeper conflicts over the size of government and its mandates. Similarly, the way in which a frame affected the policy process was context specific, but research showed framing influences in variation from great detail (*n* = 33), to some detail (*n* = 15), to little or no detail (*n* = 3).

A number of respectable framing articles from political psychology and communication were excluded from this review of the health literature for two reasons. First, this body of work was focused on identifying the ways in which the media frames health issues, such as obesity (Barry *et al.* 2011; Gollust *et al.* 2013; Niederdeppe *et al.* 2014). Many of these articles did not assess how specific health policies, programmes or legislation was framed, but rather how disease or problems are socially constructed by the media. Second, these articles frequently focused on how framing affects public opinion. The authors often mentioned that public opinion affects policy, but this was not the explicit focus of these studies. Content analysis, a method of analysing media discourse, was well-represented in 52 selected articles, but only because these showed how media constructions affected the health policy process. To suggest that the media shapes public opinion, which in turn affects policy, was considered insufficient to address our main research question and be included in the final review.

## Discussion

Descriptively, the results of this scoping review suggest that the research on framing in health is somewhat limited. First, compared with the large number of articles that mentioned framing, there are relatively few studies that focus specifically on the ways in which ideas and policies are framed. Second, this lack of framing research is accentuated when looking geographically and thematically. The bulk of framing research has historically been conducted in North America and Europe on a small set of health issues such as infectious disease control and the regulation of harmful substances. Third, most framing research has been conducted by social scientists, with considerably less situated within health policy departments or published by health policy journals. This skew, in geographic, thematic and disciplinary focus, is possibly explained by rationalist hegemony in industrialized countries as much as by simple disciplinary capture.

In addition to a descriptive overview of the scope of framing research, this review generated many analytical insights. The central goal of this review was to determine what is known from the existing literature about the influence of frames and framing on the policy process. The short answer is that quite a lot is known about a few issues in a few contexts. A more nuanced interpretation of the findings; however, points to several areas that require in-depth explanation to identify strengths and shortcomings of the existing research. This involves an appraisal by the review’s authors of what constitutes insightful framing research and what constitutes somewhat underdeveloped framing research.

First, it is important to revisit the underlying purpose of framing research. Much framing research operates from a constructivist epistemology that contests the view that knowledge is an objective, knowable and measureable entity which exists independently of the researcher and the research process. The theoretical basis of a discursive mode of policy analysis associated with framing research is derived from Critical Theory and Post-modernism. Following Habermas’s theory of communicative rationality, reason is located in the structures of interpersonal communication rather than the natural world (Habermas 1985). Similarly, Foucault emphasized that power cannot be possessed but is exercised through knowledge and discourse, which serve as a form of social control (Foucault 1980). Although Habermas and Foucault differed significantly in their understandings of the social world, their work provides the intellectual foundation of interpretive policy analysis (Fischer 2003). As such, interpretive research on framing looks at how actors create meaning in the policy process and how they package these meanings for instrumental and expressive purposes. In this way, a frame emerges, interacts with others and helps shape the terrain of the debate. Framing research does not predict change or advocate for a particular way of seeing the world. Instead, it seeks to provide an explanation for human behaviour in the policy process and how this collectively structures subsequent interactions. To use Goffman’s original conception (1974), framing is useful for understanding, ‘What is going on here?’ It enables actors (and policy analysts) to make sense of daily experience, understand a problematic situation, organize experience and act in particular way (Goffman 1974).

The scoping review was partially successful in answering the original question of what is known about the way frames and framing influences the policy process in the health sector. On the one hand, a great deal is known about highly contextualized debates over a narrow set of health issues. On the other hand, the body of scholarship on framing research offered relatively little internal coherence. This suggests that the interdisciplinary nature of framing research presents a challenge for both the reviewer and a review methodology native to biomedicine. Nevertheless, a few strong themes emerge and are reflected in Appendix, which surveys the 52 included articles.

First, some issues, such as environmental management, may not appear to be ‘health’ issues, but through policy deliberation, are framed as such (Iannantuono and Eyles 2000). This raises questions about the exclusivity of the health policy process. Many articles illustrate that policymaking is an expansive process that transcends issue domains and involves deliberation from multiple segments of society. In this way, social problems such as homelessness (Noy 2009), injection drug use (Berger 2013), violence (Dodge 2008), environmental hazards (Frickel 2004) and assisted reproductive technologies (L’Espérance 2013) can gain political support by being reframed as ‘health’ issues.

Second, a variety of theories and methods can be used to interpret the influence of frames on health policy. Though theory tends to reflect framing research’s multiple disciplinary lineages, common to most studies was a strong constructivist epistemology. Although a variety of methods were employed for analysis, most articles relied on a similar set of data sources, including some combination of interview transcripts, media transcripts and an array of different documents from legislative briefs to organizational position articles. To adequately describe the effects of frames on the policy process, most articles were qualitative, though many of the media analyses involved quantitative analysis of a frame’s usage over time.

Third, articles that presented multiple frames provided a more convincing assessment of its influence on policy than articles that described the evolution of a single frame over time. The reviewers, who were uniformed about the substantive issues in the identified articles prior to conducting the review, found it much easier to identify the interplay of ideas in the policy process, when there was a moderate amount of organized frames. But, in framing (as in life) more is simply not better. More important than the quantity of frames, was the way in which the authors organized them either hierarchically or based on established theory. In this way, careful analysis of the evolution of a single mental health collective action frame in Scotland proved insightful (Sturdy *et al.* 2012). In another example, it was relatively easy to follow research into the framing of contraceptive decisions because the authors showed how two ‘inclusive’ frames interacted with three ‘exclusionary’ frames (Rasmussen 2011). Even when a larger number of frames were represented, as in Roth *et al.*’s (2003) work on tobacco, the interaction among them was easy to follow because the authors organized frames into master (*n* = 1), diagnostic (*n* = 1), prognostic (*n* = 3) and counter (*n* = 5) frames, based on Benford and Snow’s (2000) typology of collective action frames. On the other hand, work on the social determinants of health that identified 44 different frames, proved cumbersome and raised as many questions as it answered (Andress 2007). This finding, that organization is possibly more insightful than revealing minute distinctions, underscores the significance of incorporating theory into framing research.

Fourth, research that embedded and internalized a range of framing research proved more insightful than research that gave little attention to theory. This finding was somewhat surprising given that the presence of framing theory served as an inclusion/exclusion criterion. In research on infectious disease (Doan and Kirkpatrick 2013) and health inequalities (Adams *et al.* 2010), the absence of framing theory is evident in the limited extent to which framing demonstrates conflict and change in the policy process. Similarly, a neo-institutionalist article (Inoue and Drori 2006) provided a sound theoretical basis for a sociological study, but an unconvincing analysis of how frames influenced the policy process. On the other hand, work on reproductive health (L’Espérance 2013), health financing (Tynkkynen *et al.* 2012), tobacco (Smith 2013b) and alcohol (Hawkins and Holden 2013) illustrate how a strong theoretical foundation on framing and the interplay of contested ideas guides the analysis. Furthermore, these studies illustrate the value of abductive reasoning, to move iteratively between empirical findings and framing theory.

Fifth, research that presented multiple actors, contested policy arenas and highly charged ideas proved to be useful in furthering our understanding of framing in health. This finding may be attributable to the fact that some disciplines, such as policy studies and political sociology, are inherently better positioned to capture the contested field than others, such as linguistics or cognitive psychology. Studies that looked at a narrow range of stakeholders, in a single domain, and fewer frames provided little account of contestation and therefore underdeveloped linkages with the policy process (Iannantuono and Eyles 1997; Moret-Hartman *et al.* 2006; Abraham 2011). Many of the articles that provided a nuanced account of contestation and change in the policy process were in longer dissertation/thesis/book formats (Andress 2007; Redington 2009; Ofori-Birikorang 2010; Berger 2013; L’Espérance 2013; Oronje 2013; Sardell 2014). This suggests that the highly contextual nature of framing research, combined with a qualitative analysis of the often-opaque forces that shape policy, is difficult to present within the confines of the journal format. This might provide a partial explanation as to why concise, coherent and comprehensive framing research appears to be in short supply in the health policy literature (given the restrictive word counts of journal articles in the field).

Based on the insights of this review, we propose a list of considerations for framing research on the policy process (see Table 3). Although this list is by no means exhaustive, nor does it favor a disciplinary approach to framing research, it should serve as an adequate launch point for discursive investigations into the role that ideas play in health policy. Furthermore, because this list has been developed based upon the evidence presented in this review, the strength or weaknesses of proposed research can be assessed based on the extent to which the endeavor accounts for these broad considerations.

**Table 3.** Considerations for conducting framing research

Consideration
• Is the research informed by framing theory?
• Is there a clear statement of epistemology?
• Are a variety of actors identified?
• Are multiple frames presented/interpreted?
• Are frames organized based on established theory?
• Are multiple levels of frame abstraction clearly distinguished?
• Is a frame sponsor identified as a participant in the process?
• Does the research demonstrate how frames evolve and conflict?
• Is there a portrayal of policy contestation as a struggle over ideas?
• Does the research explain why some frames prevail and others fail?
• Is there a clear influence of framing on the policy process?

There are several important findings from this review that further our understanding of frames and point to directions for strengthening their analysis across disciplines. First, there was a lack of clarity between framing analysis as theory and method in the health literature. In fact, frame or framing analysis seems to mean different things to different researchers, depending largely on their disciplinary focus. Many articles drew on the concept of framing as the basis for an empirical research project in which various themes were identified, labeled as frames, and contradictions between frames were described. Other articles, used a range of analytical techniques, identified as frame analysis, to systematically work through the discursive elements of a given text or speech act. This methodologically oriented frame research included a popular form of content analysis based on Entman’s four framing functions as well as a method for identifying the linguistic artifacts of frames using Gamson’s signature matrix. Though the indiscriminate use of framing as both theory and method might seem problematic for defining the boundaries of a research paradigm, it also represents a potential strength of framing research. Creed *et al.* (2002), further elaborate, ‘Because of its underlying attention to context, standing, and power, frame analysis provides us with a linked theory and methodology that gets us farther in our projects than other methodologies’ (Creed *et al.* 2002). To be fair, many articles did make mention of some type of framing theory and implied that the methods were a form of frame analysis, but the most insightful studies were those that used abductive reasoning to move iteratively between empirical findings and framing theory.

Second, despite attempts to develop frame analysis as a research paradigm, the health policy literature suggests a lack of consensus exists across disciplines. Efforts to bring conceptual clarity to framing research have come from the fields of political communication (Entman 1993; Pan and Kosicki 1993; Scheufele 1999; Scheufele and Iyengar 2012), political psychology (Druckman 2011) and policy studies (Van Hulst and Yanow 2014). This review suggests that these endeavours have yet to produce a coherent and unified corpus of framing research in the health policy literature. Nevertheless, the review illustrates that framing research is an important form of policy analysis and that it is distinct from ‘simple researcher-designated labels’ (Kosiki 1993). We contend that researchers interpret and deploy the concept of frames (and the process of framing) in particular ways. Yet, this contention is in keeping with a constructivist epistemology.

One goal of this review was to use framing research as a vehicle to marry the health policy literature with the wider policy studies scholarship. The rationale for using frames, as an ideational approach, is that by nature framing is interdisciplinary and its use as both theory and method is gaining credence. This review suggests the same is true both quantitatively and qualitatively in the health policy literature. For example, the ‘evidence-based’ literature is increasingly looking to ideational approaches to analysing complexity in decision making (Smith 2013a). Another example, a widely cited framework for assessing the generation of political priority in health, makes use of ‘internal’ and ‘external’ frames (Shiffman and Smith 2007). This is analogous to ‘coordinative’ and ‘communicative’ discourse, as advocated by a new brand of discursive institutionalist scholarship in political economics (Schmidt 2008). This indicates that some ideas are beginning to enter mainstream modes of policy analysis in the health sector, but it also points to some differences. Although the two forms of discourse in institutionalist scholarship are integrated into a highly contextualized way of looking at the discursive interplay of policy ideas, in the health policy framework, they are positioned as 2 variables amongst 12 that must be considered in explaining why something happens (Shiffman and Smith 2007). The argument by ideational scholars is not that ideas or frames are an ingredient in bringing about change; rather they represent the causal beliefs that bring change about (Béland and Cox 2011). The policy studies literature on framing emphasizes the primacy of ideas and an adequate analysis of them would take into account other salient aspects included in the Shiffman and Smith framework (2007) such as actor power, political context and issue characteristics. This shift in emphasis is manifest in the applications of the health policy framework, which is biased in favor of a deductive mode of proving or testing theories about why some things happen (Walt and Gilson 2014). A mode of analysis that focuses to such a limited degree on frames often raises more questions, particularly with respect to the irrational nature of decision making, than it actually answers. By looking at the way in which the articles included in this review are structured, the intentions of the researchers writing them, and what they are trying to achieve, we shift the nature of the discussion around policy analysis in health. Similarly, by looking at the scope of framing research in one issue domain, such as health, insights may be generated to further broader policy studies scholarship on framing.

## Limitations

The limitations of this review are multiple. The body of evidence proved difficult to corral given the abstract nature of the subject material and the systematic nature of the scoping review framework. This ranged from the relatively simple tasks of defining categories for strains of disciplinary background, theory and methods to distinguishing amongst more abstract characteristics of the articles such as epistemology, evidence of contestation and demonstrated affect on the policy process. Similarly, the inclusion/exclusion criteria were such that it resulted in cursory abstract review of a large number of articles, which may have led to some articles being unfairly excluded. Further, by including articles with a strong theoretical basis, we excluded various strands of relevant framing research, including experimental findings germane to behavioural economics and media analyses from political psychology, discourse studies and communications research. These articles were largely excluded because they showed little or no direct bearing on the policy process. Still, they remain important and under-represented dimensions of framing research.

## Conclusions

This scoping review demonstrates the potential of framing research as a means of understanding the influence of ideas and human behaviour in the policy process. Despite a relative paucity of data for many health issues, demonstrable policy struggles occur in a variety of contexts for a few health issues such as tobacco control and pandemic influenza preparedness. By framing ideas in a particular way, actors evoke deeply held values that shift the terrain of the debate, transforming social phenomena into problems, implying a set of solutions, forming coalitions of interest and mobilizing specific policy responses. More research should be conducted, particularly in LMICs, to gain a better understanding of the complex policy terrain in the health sector.

The scoping review was a useful approach for harnessing the diverse pool of evidence located on the periphery of traditional health policy research. As a relatively new methodology and perhaps an unfamiliar body of theory, framing research has yet to receive adequate attention in the health literature. The analytical insight generated by the 52 articles included in this review was quite variable with framing approaches reflecting distinct research traditions. This article contributes to the wider (non-health) policy literature on framing by identifying several features of insightful framing research. In this way, we hope to strengthen the health sector’s contribution to the policy studies literature while positioning framing research as an important vehicle for understanding human behaviour in the health policy process and ultimately leading to a deliberative mode of policy analysis that contributes to the shared goal of health systems strengthening.

## Appendix

**Appendix:** Overview of selected research

Author, Year	Journal	Country	Type	Method	Data source	Health issues	Frames	Contestation	Affect on policy process
Abraham (2011)	*Political Studies*	Global	International relations	Historical media analysis	Print media	Infectious disease—Avian influenza	(1) Security	Little	Some
Adams *et al.* (2010)	*Gay and Lesbian Issues and Psychology Review*	Multiple	Psychology	Discourse analysis	Documents, reports	Inequalities—LGBT health	(2) Biomedical, biopsychosocial	Yes	Little
Andress (2007)	*Ph.D. Thesis*	UK	Political science	Signature matrix	Media, newspapers	Social determinants	44 different frames	Yes	Some
Berger (2013)	*Ph.D. Thesis*	USA	Policy studies	Case study	Interviews issue papers, newspaper, testimony	Substance misuse—injection drug use	(4) Moral, political, scientific, other	Yes	Yes
Blackman *et al.* (2012)	*Sociology of Health and Illness*	UK	Health policy	Case study	Interviews	Health inequalities	(4) Politics, audit, evidence, treatment	Yes	Yes
David *et al.* (2012)	*Population Research and Policy Review*	Phillipines	Policy studies	Discourse analysis, network analysis	Legislation, documents	Reproductive health—demography	(6) Development, population management, reproductive health, vs abortion, moral values, anti-familyplanning/anti-abortion	Yes	Yes
Daw *et al.* (2014)	*Journal of Health Politics, Policy and Law*	Canada	Health policy	Content analysis	Newspapers,	access to medicines—expansion of drug/pharmacy benefits in national plan	(25) problem frames: values-related (w4 sub-frames), cost-related (w4 sub-frames), other; policy solutions: (3 sub-frames) policy options: (8 sub-frames); barriers to policy: (6 sub-frames)	Yes	Yes
Doan and Kirkpatrick (2013)	*Policy Studies Journal*	USA	Political science	Content analysis	Newspapers	Infectious disease—HPV	(4) politics, public health, economic, morality	Yes	Some
Dodge (2008)	*American Psychologist*	USA	Psychology	Strategic frame analysis	Unclear	Violence	(7) superpredator, moral defect, quarantine, man as computer, corrective surgery, vaccine, chronic disease	Yes	Some
Driedger and Eyles (2003)	*Social Science and Medicine*	Canada	Policy studies	Frame analysis	Interviews, newspapers	Environmental Health—water quality	(5) Voluntary vs involuntary risk, chlorination disinfection saves lives; with 3 sub-frames: luxury of the first world, balancing risks, single bad actor vs complex mixture vs chlorine byproducts cause cancer	Yes	Yes
Esmail and Kohler (2012)	*Globalization and Health*	Canada	Policy studies	Content analysis, critical realist evaluation	Legislative transcripts, legislation	Access to drugs	(4) liberty, equity, efficiency, security	Yes	Yes
Firbank (2011)	*Journal of Aging Studies*	Canada	Policy studies	Frame-critical policy analysis	Documents Government papers	Population health—geriatrics	Many: Moral, Dominant institutional action frames, and dominant policy frames	Some	Yes
Fogarty and Chapman (2011)	*Drug Alcohol Review*	Australia	Health policy	Content Analysis, Frame Analysis	Newspapers	Substance misuse—alcohol control	(4) 2 in favor of alcopops tax (consumption reduction, loophole) and 2 against (substitution, revenue raising)	Some	Some
Fogarty and Chapman (2012)	*BMC Public Health*	Australia	Health policy	Content analysis, frame analysis	Newspapers	Substance misuse—alcohol control	(10) News media frames supportive of adv. restrictions (5) and not supportive of adv. restrictions (5)	Some	Some
Frickel (2004)	*Social Problems*	Global	Sociology	Frame analysis	Documents, interviews	Environmental health—toxicology	(2) chemical risk became genetic hazard	Some	Some
Garvin and Eyles (2001)	*Social Science and Medicine*	Multiple	Health policy	Case study	Documents, interviews	NCDs—cancer (skin)	(Many) Table 1 - communicator, text, receiver, culture - narratives	Yes	Some
Goss (1996)	*Scandinavian Journal of Management*	UK	Sociology	Frame analysis	Documents	Infectious disease—HIV/AIDS	(2) Defensive - threat to organizational success, constructive - medical problem	Some	Some
Hawkins and Holden (2013)	*Critical Policy Studies*	UK	Policy studies	Frame-critical policy analysis	Documents, Interviews	Substance misuse—alcohol	(5) problem restricted to a minority vs societal problem, a public health issue, pricing vs anti-pricing prescriptions	Yes	Yes
Iannantuono and Eyles (2000)	*Environmental Management*	Multiple	Policy studies	Frame-critical policy analysis	Reports	Environmental health	(Many) by level of framing: action frames - managing ecosystems, changing human behavior	Yes	Yes
Iannantuono and Eyles (1997)	*Social Science and Medicine*	Canada	Policy studies	Semiotic schemata	Documents	Health systems	(Many) Various components labeled as signs or codes	Little	Some
Ibrahim (2007)	*Crossroads*	Singapore	Policy studies	Content analysis	Government Press releases	Infectious disease—SARS	(1) War rhetorical frame/gov., action frame/policymakers	Some	Yes
Inoue and Drori (2006)	*International Sociology*	Global	Sociology	Organization assessment	Unclear	Global health governance	(4) International health as charity, professional activity, means for development, basic human right	Little	Llittle
Jenkin *et al.* (2011)	*Obesity Reviews*	New Zealand	Health policy	Case study, signature matrix	Submissions to Parliament Inquiry	NCDs—obesity	(Many) Table 3: by position (7), causal roots (6), solutions (3), and core values (2 w/8sub-frames)): market justice vs social justice	Yes	Some
Johnson (2010)	*Global Public Health*	Honduras	Health policy	Content analysis	Documents	Health systems	(2) Economic liberalization, distributional equity,	Yes	Unclear
Kamradt-Scott (2012)	*Global Public Health*	Global	International relations	Case study	Documents, interviews	Infectious disease—influenza	(1) Evidence-based medicine as an emergent frame	Some	Some
Kamradt-Scott and McInnes (2012)	*Global Public Health*	Global	International relations	Case study	Documents, interviews	Infectious disease—influenza	(1) Security	Yes	Yes
Kolker (2004)	*Sociology of Health and Illness*	USA	Sociology	Frame analysis	Congress testimony, media	NCDs—cancer (breast)	(5) reframing breast cancer from “private problem” to “public health problem” culturally resonant frames: as epidemic, as gender equity problem, as threat to families	Some	Yes
Kwan (2009)	*Sociological Inquiry*	USA	Sociology	Frame analysis, signature matrix	Documents	NCDs—obesity	(Many) Cultural frames: medical, social justice, market choice w/ sub-frames embedded in matrix	Yes	Some
L’Espérance (2013)	*Ph.D. Thesis*	Multiple	Policy studies	Interpretive policy analysis	Documents, Interviews, Newspapers	Reproductive health—technologies	(6) Moral, Medical, Administrative, Legal, Family-building, Experience-based	Yes	Yes
Menashe and Siegel (1998)	*Journal of Health Communication*	USA	Media studies	Signature matrix	Newspapers	Substance misuse—tobacco control	(21) 11 tobacco interest frames, 10 tobacco control frames (arguments) - 6 industry frames and 4 advocate frames (mapped principles/values)	Yes	Some
Moret-Hartman *et al.* (2006)	*Health Policy*	Netherlands	Health Policy	Argumentative policy analysis	Interviews	Health systems—service delivery (prescribing practices)	(Many) > 16, organized in a interpretive matrix	Little	Some
Noy (2009)	*Social Problems*	USA	Sociology	Frame, network, and content analysis, Participatory action research	Documents, Interviews, Media, participant observation	Social determinants—homelessness	(4) Master frames: individual, systemic, social control, bureaucratic failure Diagnostic—individual, structural	Yes	Yes
Ofori-Birikorang (2010)	*Ph.D. Thesis*	Ghana	Media studies	Ethnographic content analysis	Newspapers, interviews	Financing—national health insurance reform	(33) 7 main news frames with 26 sub-frames	Yes	Yes
Oronje (2013)	*Ph.D. Thesis*	Kenya	Policy studies	Case study	Documents, interviews, participant observation	Reproductive health—sexual/reproductive health	(Many) SRH as a moral, cultural, medical, and human rights narrative…multiple frames included within each narrative	Yes	Yes
Parkhurst (2012)	*Evidence and Policy*	US policy in Uganda	Health policy	Discourse analysis	Documents, interviews, texts	Infectious disease—HIV/AIDS	(2) sexuality, morality	Yes	Yes
Parkhurst and Vulimiri (2013)	*Global Public Health*	Global	Health policy	Review	Unclear, research, reports	NCDs—Cancer (Cervical)	(3) NCDs umbrella, women’s right and health, co-morbidity of HIV/AIDS	Yes	Some
Paterson and Marshall (2011)	*Journal of Canadian Studies*	Canada	Policy studies	Frame analysis	Newspapers	Health systems—workforce (Midwifery)	(4) Ontario (3): progress (metaframe), legal, boundary (issue frames); Quebec (1): boundary	Yes	Yes
Peters *et al.* (2013)	*Journal of the International AIDS Society*	Global	Health policy	Discourse analysis	Documents	Infectious disease——HIV/AIDS	(4) Sexuality (main) - also gender, reproductive rights, sexual rights	Yes	Yes
Rasmussen (2011)	*Administration and Society*	USA	Policy studies	Interpretive policy analysis	Legislative testimony, letters, statements	Reproductive health—contraception	(5) Incl. frames (2): Medical, gender/class based equity Excl. frames (3): market-based, religious, elective/immoral procedure	Yes	Yes
Redington (2009)	*Ph.D. Thesis*	Untied States	Health policy	Mediated thematic analysis	Documents, Congress hearings, interviews	Access to drugs and medications	(4) ODA Reform as Economics and Access, Patient Relief, Rules of Participation, Congressional Action	Yes	Yes
Reubi (2012)	*Global Public Health*	Global	International relations	Case study	Documents, interviews	Substance misuse—tobacco control	(1) Human rights	Yes	Yes
Rohlinger (2002)	*The Sociological Quarterly*	USA	Sociology	Content analysis	Print media	Reproductive health—decisions	(2) abortion framed as (constitutional) right vs morality	Yes	Yes
Roth *et al.* (2003)	*Social Studies of Science*	USA	Sociology	Frame analysis, content analysis	Federal Regulation, Public Response	Substance misuse—tobacco control	(>10) master frame: science (1) diagnostic frames: preventable illness, (3) prognostic frames: reducing access, reducing appeal, educating youth about health risks; (5) counter-frames - scientific, ideological, economic, political, procedural (all with sub-frames)	Yes	Yes
Rushton (2012)	*Global Public Health*	Global	International relations	Case study	Documents, interviews	Infectious disease—HIV/AIDS	(3) public health security/safety, economic re-framing to a single human rights based frame	Yes	Yes
Saguy and Riley (2005)	*Journal of Health Politics, Policy and Law*	USA	Sociology	Case study	Documents, interviews, participant observation	NCDs—obesity	(5) Body diversity, risky behavior, disease, epidemic, illness	Yes	Some
Sardell (2014)	*Book*	USA	Political science	Case study	Documents interviews, previous research	Financing—health insurance reform	(3) Preventable/solvability, cost-effective, human capital	Yes	Yes
Smith (2013b)	*Social Policy and Administration*	UK	Policy studies	Review	Documents: histories, research	Substance misuse—tobacco control	(4) Health-oriented, free personal choice, economic, reducing health inequalities	Yes	Yes
Studlar (2008)	*The Review of Policy Research*	USA	Political science	Historical analysis	Unclear	Substance misuse—tobacco control	(6) Public health, political economy, morality; good vs evil, social hygiene, tax grab (re-frames)	Yes	Yes
Sturdy *et al.* (2012)	*Social Policy and Administration*	Scotland	Sociology	Frame analysis	Documents, interviews, participant observation,	Mental health	(1) Mental well-being (collective action frame)	Yes	Yes
Tynkkynen *et al.* (2012)	*BMC Health Services Research*	Finland	Health policy	Frame analysis, signature matrix	Interviews	Financing—strategic purchasing	(5) Rational reasoning, pragmatic realism, promoting diversity of providers, benefits for the municipality, good for local people.	Yes	Yes
Williams 2012	*Global public health*	Global	International relations	Case study	Documents, interviews	Access to medicines	(5) Dominant economic framing vs counter frames of human rights, negative consequences for access, undermining global public goods, and negative impact on development	Yes	Yes
Woodling *et al.* (2012)	*Global Public Health*	Global	International relations	Case study	Documents, interviews	Infectious disease—HIV/AIDS	(2) frame shift from ‘AIDS to development’ to ‘AIDS and development’	Yes	Yes
